# Elastic Electron Scattering from Methane Molecule in the Energy Range from 50–300 eV

**DOI:** 10.3390/ijms22020647

**Published:** 2021-01-11

**Authors:** Jelena Vukalović, Jelena B. Maljković, Karoly Tökési, Branko Predojević, Bratislav P. Marinković

**Affiliations:** 1 Institute of Physics Belgrade, University of Belgrade, Pregrevica 118, 11080 Belgrade, Serbia; jelena.vukovic@pmf.unibl.org (J.V.); jelenam@ipb.ac.rs (J.B.M.); 2 Faculty of Science, University of Banja Luka, Mladena Stojanovića 2, 78000 Banja Luka, Republic of Srpska, Bosnia and Herzegovina; bpredojevic@teol.net; 3 Institute for Nuclear Research (ATOMKI), 4026 Debrecen, Hungary; tokesi@atomki.hu

**Keywords:** methane, cross-section, elastic scattering, electrons

## Abstract

Electron interaction with methane molecule and accurate determination of its elastic cross-section is a demanding task for both experimental and theoretical standpoints and relevant for our better understanding of the processes in Earth’s and Solar outer planet atmospheres, the greenhouse effect or in plasma physics applications like vapor deposition, complex plasma-wall interactions and edge plasma regions of Tokamak. Methane can serve as a test molecule for advancing novel electron-molecule collision theories. We present a combined experimental and theoretical study of the elastic electron differential cross-section from methane molecule, as well as integral and momentum transfer cross-sections in the intermediate energy range (50–300 eV). The experimental setup, based on a crossed beam technique, comprising of an electron gun, a single capillary gas needle and detection system with a channeltron is used in the measurements. The absolute values for cross-sections are obtained by relative-flow method, using argon as a reference. Theoretical results are acquired using two approximations: simple sum of individual atomic cross-sections and the other with molecular effect taken into the account.

## 1. Introduction

Methane (CH_4_) is the simplest alkane. Its molecule has tetrahedron shape, belongs to the T_d_ point group symmetry, and does not possess dipole and quadrupole moments. Furthermore, methane is widely distributed in the Solar System. In general, the inner planets Mercury [1], Venus [2], and Mars [3] are methane-poor, except Earth, whereas outer planets Jupiter [4], Saturn [5], Uranus [6], and Neptune [7] have methane-rich atmospheres. Currently, the methane levels in Earth’s atmosphere are around 1.6–1.8 ppmv and are considered one of the main causes of the greenhouse effect. Greenhouse effect caused by methane is about eight times that of CO_2_ [8]. Emission of CH_4_ in atmosphere is about 40% from natural, and about 60% from anthropogenic sources (agriculture, energy, and waste sectors [9]). In past decades methane’s growth rate was changing, and in recent years, it has been increasing [10]. That is a reason why investigation of this gas again becomes consequential.

Electron collisions with methane are very important in plasma physics. In mixture with hydrogen and argon, CH_4_ is used for r. f. plasma-enhanced chemical vapor deposition (R. F. PECVD) of nanocrystalline diamond films [11]. On the other hand, methane is less used than silane in design of solar cells. Its use in the preparation of amorphous SiC *p*-layers is most often emphasized in a PECVD process using high R.F. power at relatively low temperature [12]. When a Tokamak is operated at high density and high temperature methane plays a dominant role in the edge plasma region [13]. Particles and energy are expelled from the plasma and are transported to the vessel wall, which leads to complex plasma-wall interactions. These interactions create impurities in the plasma, including methane and its derivates, resulting in significant cooling of plasma, which can prevent achievement of rector relevant conditions. Therefore, understanding transport features of methane in plasma and interpretation of electron methane collisions in different physical processes plays a significant role in laboratory research in different fields of physics as well as in the investigation of properties atmospheres planets in Solar System. Finally, from a theoretical perspective and, because of its simplicity, methane can serve as a test molecule for advancing novel electron-molecule collision theories (e.g., Blanco et al. [14], Allan [15]).

Elastic electron scattering from methane molecule has been studied intensely in the past, and the most recent review of elastic differential cross-sections is given in [16]. Recommended set of data for electron/methane interactions is given by Song et al. [17]. For low incident electron energies (below 50 eV), elastic electron scattering from methane molecule has been studied experimentally and theoretically equally extensively. To cite few most recent papers, Allan [15] measured absolute differential elastic (impact electron energies 0.4–20 eV) and vibrational excitation cross-sections using an electron spectrometer with a magnetic angle changer, which allows measurements of differential cross-sections (DCSs) for backward angles. Bettega et al. [18] calculated DCSs and integral cross-sections (ICSs) for energies between 3 and 10 eV, using Schwinger multichannel method with pseudopotentials. Bundschu et al. [19] presented both experimental and theoretical results; measurements of DCSs using crossed beam apparatus and relative flow method, with He as a reference gas, and calculations using a body-fixed, single-center for close-coupled equations. Shyn and Cravens [20] reported DCSs for methane in energy range from 5 to 50 eV, using modulated crossed-beam method and He as reference gas for normalizing relative data to the absolute scale. At even lower electron energies, from 0.1 to 1.8 eV, an important study was one by Sohn et al. [21] where the references from even earlier measurements and calculations can be found. Several authors used R-matrix method to calculate cross-sections form methane [22,23,24].

As for papers that contain data for DCSs at intermediate-to-high energies (E_0_ ≥ 50 eV); Boesten and Tanaka [25] reported measured DCSs (electron energies between 1.5 and 100 eV), ICSs, and momentum transfer cross-sections (MTCSs) for methane. Measurements were done using crossed electron and molecular beam technique where observed DCSs were normalized point by point with the help of simultaneous measurements of DCSs of He. Vušković and Trajmar [26] obtained relative cross-sections for 20, 30, and 200 eV impact energies and normalized them to the absolute measurements of Tanaka et al. [27] for 20 and 30 eV, and to the calculations of Dhal et al. [28] for 200 eV. Cho et al. [29] published measured data for DCSs (ICSs and MTCSs as well) for electron elastic scattering from methane over scattering angles between 10° and 180° for incident electron energies from 5 to 100 eV using crossed beam spectrometer combined with a magnetic angle-changing device. Relative flow with He was exploited for normalization. They used the iterative Schwinger variational method combined with distorted-wave approximation to solve scattering equations. Sakae et al. [30] measured DCSs using crossed-beam method in angular range 5–135° for electron energies 75–700 eV. Relative DCSs were converted to the absolute values at 30° by using the ratio of elastic DCSs of the target gas to that of He. Iga et al. [31] used crossed beam apparatus to obtain scattering intensities (100–500 eV incident electron energies), which were converted to the absolute scale using relative flow method (Ne was used as a reference gas). Also, they used Schwinger variational method combined with the distorted-wave approximation to study elastic electron scattering (1–500 eV) theoretically. Jain [32] used a spherical optical complex potential model to investigate electron interaction with methane over a wide energy range from 0.1 to 500 eV. Mahato et al. [33] obtained analytical expressions for the static potentials of electron scattering from methane using Gaussian wave functions and studied elastic scattering from 10 to 500 eV incident electron energies utilizing those static potentials along with exchange and polarization potentials. Song et al. [17] presented recommended elastic DCSs and ICSs obtained by averaging other authors’ data [15,19,20,21,25,29,30,31]. List of experimental and theoretical work on DCS for elastic electron scattering from methane molecule, in energy range of our interest is shown in Table 1. For the present work, the paper by Fuss et al. [34] is interesting because they provided the recommended set of data for differential and integral cross-sections for methane, including elastic electron scattering. They obtained their dataset by merging and averaging other authors’ data [19,25,30,33] for lower energies and calculating ones for high energies (40–10 keV).

In this paper, theoretical and experimental results for elastic electron scattering from methane are shown. Obtained data include absolute differential cross-sections (DCSs) for elastic scattering for the incident electron energy range from 50 to 300 eV (with 50 eV steps) and angular range from 5° to 125° (with 5° steps), integral cross-sections, and momentum transfer cross-sections (ICSs and MTCSs, respectively) for every measured energy. The experiment was performed on a crossed-beam apparatus. As in most previous experiments [19,20,29,30,31], relative intensities were put on the absolute scale with help of relative flow method. The difference was in reference gas; in almost all experiments, He was used (except for Iga et al. [30] who used Ne), in our argon was reference gas. Theoretical results were obtained by calculating atomic cross-sections for molecular components, with two approximations used for molecular cross-sections simulations; simple sum (model 1) of atomic cross-sections and with molecular effect taken into the account (model 2). The existing variety of cross-section datasets for methane reflects our motivation to perform this study and, at the same time, to provide data at some of the impact energies where there are no previously measured data or where data require an independent confirmation. By exploiting different reference gas, Ar in this case, and performing new calculations that are using a coherent sum of atomic wave functions, we provide the independent and excessive datasets of cross-sections for this important molecule.

The paper is organized as follows. Theory and calculations of DCSs are explained in Section 2. Experimental setup and measurement procedure are given in Section 3. The obtained results are listed in Table 2 and presented graphically, including the comparison with the existing experimental and theoretical data, all given in Section 4. A discussion is given in Section 5. Finally, Section 6 is reserved for the conclusion.

## 2. Theory

In this work, the key to the determination of the elastic cross-section calculations of CH_4_ is the atomic cross-section calculations for the components of the molecule. We assume for the determination of the effective interaction at distance **r** between a projectile electron and the target that the scattering potential can be expressed as:

(1) $$V\left(r\right)=V_{\mathrm{st}}\left(r\right)+V_{\mathrm{ex}}\left(r\right)+V_{\mathrm{cp}}\left(r\right),$$

where *V*_st_ is the electrostatic potential, *V*_ex_ is the electron exchange potential and *V*_cp_ is the correlation-polarization potential. The electrostatic potential for the interaction between an electron and the target atoms:

(2) $$V_{\mathrm{st}}\left(r\right)=-e\left[\varphi_{n}\left(r\right)+\varphi_{e}\left(r\right)\right],$$

where *φ*_n_ and *φ*_e_ are respectively the components of nucleus and the electron cloud of electrostatic potential. 

The Furness-McCarthy exchange potential [35] is used for the electron exchange potential:

(3) $$V_{\mathrm{ex},\mathrm{FM}}\left(r\right)=\frac{1}{2}\left[E-V_{\mathrm{st}}\left(r\right)\right]-\frac{1}{2}{\left\{{\left[E-V_{\mathrm{st}}\left(r\right)\right]}^{2}+4\pi a_{0}e^{4}\rho_{e}\left(r\right)\right\}}^{1/2}.$$

An accurate correlation-polarization potential combines the long-range polarization potential, *V*_cp,B_(*r*), with the correlation potential *V*_cp_(*r*) obtained from the local-density approximation (LDA) and it can be expressed as [36]:

(4) $$V_{\mathrm{cp},\mathrm{LDA}}\left(r\right)\equiv \{\begin{array}{ll} \max\left\{V_{\mathrm{cp}}\left(r\right),V_{\mathrm{cp},B}\left(r\right)\right\}, & r<r_{\mathrm{cp}};\\ V_{\mathrm{cp},B}\left(r\right), & r\geq r_{\mathrm{cp}}. \end{array}.$$

where *r*_cp_ is the outer radius at which *V*_cp_(*r*) and *V*_cp,B_(*r*) cross. The *V*_cp,B_(*r*) when the projectile is far from the atom can be approximated by the Buckingham potential as:

(5) $$V_{\mathrm{cp},B}\left(r\right)=-\frac{\alpha_{d}e^{2}}{2{\left(r^{2}+d^{2}\right)}^{2}},$$

where *α_d_* is dipole polarizability of the target atom and *d* is a phenomenological cut-off parameter that serves to prevent the polarization potential from diverging at *r* = 0. The experimental values of the atomic dipole polarizabilities from [37] are usually used in Equation (5). Perdew and Zunger [38] proposed a parameterization of the *V*_cp_(r) correlation potential in the following form:

(6) $$V_{\mathrm{cp}}\left(r\right)=\{\begin{array}{ll} -\frac{e^{2}}{a_{0}}\left(0.0311\ln r_{s}-0.0584+0.00133r_{s}\ln r_{s}-0.0084r_{s}\right), & r_{s}<1;\\ -\frac{e^{2}}{a_{0}}\frac{0.1423+0.1748r_{s}^{1/2}+0.0633r_{s}}{{\left(1+1.0529r_{s}^{1/2}+0.3334r_{s}\right)}^{2}}, & r_{s}\geq 1. \end{array}$$

where

(7) $$r_{s}=\frac{1}{a_{0}}{\left[\frac{3}{4\pi \rho_{e}\left(r\right)}\right]}^{1/3}$$

is the radius of the sphere that contains (on average) one electron of the gas, in units of the Bohr radius *a*_0_.

For the theoretical determination of the elastic cross-sections we used the Mott’s differential cross-section [39],

(8) $$\frac{d\sigma_{e}}{d\Omega }={\left|f\left(\theta \right)\right|}^{2}+{\left|g\left(\theta \right)\right|}^{2},$$

where *θ* is scattering angle, *f*(*θ*) and *g*(*θ*) are the spin-up and spin-down scattering amplitudes. The *f*(*θ*) and *g*(*θ*) can be expressed as:

(9) $$f\left(\theta \right)=\overset{\infty }{\underset{l=0}{\sum }}F_{\ell }P_{\ell }\left(\cos\theta \right),$$

(10) $$g\left(\theta \right)=\overset{\infty }{\underset{l=0}{\sum }}G_{\ell }P_{\ell }^{1}\left(\cos\theta \right),$$

where *P_ℓ_*(cos*θ*) are the Legendre polynomials, *P_ℓ_*^1^(cos*θ*) are the associated Legendre functions. The *F_ℓ_* and *G_ℓ_* can be calculated according to following relations:

(11) $$\;F_{\ell }=\frac{1}{2ik}\left\{\left(\ell +1\right)\left(e^{2i\delta_{\ell }^{+}}-1\right)+\ell \left(e^{2i\delta_{\ell }^{-}}-1\right)\right\};\;$$

(12) $$G_{\ell }=\frac{1}{2ik}\overset{\infty }{\underset{\ell =1}{\sum }}\left\{e^{2i\delta_{\ell }^{-}}-e^{2i\delta_{\ell }^{+}}\right\}\;,$$

where *δ_ℓ_^+^* and *δ_ℓ_^−^* are spin up and spin down phase shifts of the *ℓ*th partial wave, and *k* is the momentum of the projectile electron, respectively.

The integration of the differential cross-section over total solid angles gives us the total elastic cross-sections in the following form:

(13) $$\sigma_{e}=\int \frac{d\sigma_{e}}{d\Omega }d\Omega =2\pi \int_{0}^{\pi }\sin\theta \left\{{\left|f\left(\theta \right)\right|}^{2}+{\left|g\left(\theta \right)\right|}^{2}\right\}d\theta .\;$$

All calculations of elastic cross-section were performed by ELSEPA [40]. We used two approximations during the simulation of the molecular elastic cross-sections. In model 1, the calculated atomic cross-sections were simply added according to the stoichiometry numbers (additivity approximation). This approximation is frequently used approximation. However, it neglects the chemical-binding and aggregation effects. The electron distribution in molecules differs from that of an isolated atom of the same element. It was shown that this difference seems to have only a weak influence on the elastic DCS [40] at projectile energies larger than a few hundred eV. The effect of aggregation, the effect when the atoms are close together, has a stronger influence on the DCS [40]. In model 2 we have taken into account the positions of the atoms in the molecule. We used a single-scattering independent-atom approximation assuming that the interaction of the projectile with each atom of a molecule is given by the free-atom potential as for model 1. To determine the molecular DCS, the scattered wave at large distances from the molecule is approximated as the coherent sum of the wave functions scattered from all atoms in the molecule. In our calculations the carbon atom is located at the origin of the coordinate system. The positions of the hydrogen atoms are located at the following coordinates expressed in units of 10^-10^ m: H_1_(0.5541, 0.7996, 0.4965), H_2_(0.6833, −0.8134, −0.2536), H_3_(−0.7782, −0.3735, 0.6692), H_4_(−0.4593, 0.3874, −0.9121). We found the significant improvement in the cross-section calculations for model 2 compared with model 1.

## 3. Experiment

Experimental results presented in this paper are obtained on apparatus with crossed beams setting; incident electrons collide with a molecular beam perpendicularly. The experimental setup is placed inside the vacuum chamber, pumped with a turbomolecular pump to a typical background pressure (no gas in the chamber) about 5 × 10^−7^ mbar. The magnetic field in the collision region is reduced by two concentric μ-metal shields inside the chamber.

Incident electrons are derived from the hairpin tungsten filament (cathode) by thermoelectronic emission. The electron beam is then extracted and focused into the interaction volume by seven electrodes of the electron gun. Electron energy can vary in the range from 40 to 300 eV and is determined by the potential difference between the filament and the last, grounded, electrode, with energy resolution about 0.5 eV. When the current through the cathode filament was about 2.22 A, the electron current, measured with the Faradays cup without gas in the chamber, was approximately 100 nA. The electron gun can be rotated around the fixed detection system in the angular range from −40° to 126°.

Atomic/molecular beam is formed by stainless-steel gas needle. The length of the needle is *l* = 40 mm and its diameter, *d* = 0.5 mm. According to Lucas [40], beam properties can be predicted and optimized. Optimum atomic beam is obtained when I(0)2(NH2), where *I*(0) is axial intensity, *N* throughput and *H* beam halfwidth, is maximum. It is shown that I(0)2(NH2)∝l2d [41]. So, the optimum beam is acquired when the ratio of square of single tube length and its diameter is maximum, which is in our case 3200. These expressions can be employed for tubes where γ=dl (true dimensionless ratio of length and diameter) is below 10 (γ < 10) and gas pressure is low enough so that the mean free path of a particle is larger or equal than *d* (λ≥d). In our experimental setup γ = 80 and λ≈d.

After the interaction with the molecular beam, scattered electrons are entering the detection system. First, they are focused and slowed down to the constant pass energy of the analyzer by the four-electrode lens. Then, they are energy-analyzed by the double cylindrical mirror analyzer (DCMA). After that, elastically scattered electrons are focused by the three-electrode lens into the detector (single channel electron multiplier).

The intensity of elastically scattered electrons from methane molecule is measured as a function of scattering angle, from 25° to 125° (in 5° steps), at given incident electron energy, from 50 to 300 eV (in 50 eV steps). Experimental parameters were adjusted so that the interaction volume was kept constant. Deviations that can occur at small angles are corrected by comparing cross-sections from Ar at given energy with other authors’ data [42,43]. During the measurements, working pressure in the chamber was about 4 × 10^−6^ mbar. For each electron energy, scattering intensities are measured at least three times. To discount background scattering contributions, a gas beam was introduced to the chamber through a side leak, away from the collision area. Scattering intensities are measured and subtracted from apparent signal for each angle.

The obtained relative DCSs are converted to the absolute DCSs using relative flow method (RFM) [44]. Briefly, the signal of scattered electrons from the target molecule is compared with elastically scattered electron intensity from reference gas, at the same scattering angle, for the same electron energy and experimental conditions. The same experimental conditions for both gasses implies that their beam profiles must be closely equal, which is acquired, according to Olander and Kruger [45], under two conditions: the mean free paths (λ) behind the gas needle for both gasses must be the same and the Knudsen number, *K_L_*, defined as λ/*l*, must be in the appropriate range, $\gamma \leq K_{L}\leq 10$. The first condition is fulfilled when pressure ratio of test molecule and reference gas is inversely proportional to the ratio of squares of their gas kinetic diameters. In this study, Ar is used as a reference, with its diameter of approximately 3.58 Å. The gas kinetic diameter of the target gas, methane is approximately 3.8 Å, which gives ratio 1.13; almost the same gas pressure behind gas needle for both gasses must be applied. As for the second condition, gas pressures were low enough so that *K_L_* is approximately equal to γ, although some studies have shown that even when *K_L_* is much lower than γ, beam profiles for most gasses can still be very alike [46,47]. Besides scattering intensities of both gasses, relative flow rate is determined by measuring pressure increase in time by admitting gas into the constant volume, while a chamber outlet was closed. 

The known absolute DCSs for Ar are taken from Ranković et al. [42] for incident electron energies 50–200 eV and 300 eV and from Williams and Willis [43] for electron energy of 250 eV. In both papers, the absolute values were derived by measurements of angular dependences of elastically scattered electrons using electron spectrometers, two 127° cylindrical electrostatic energy analyzer in [43], and a double cylindrical mirror analyzer in [42] but employing different normalization procedures. While in [42] the relative flow method with He as a reference gas was used, in [43] a phaseshift analysis of the relative angular distributions of electrons elastically scattered in the energy region of the resonances ^2^P_3/2,1/2_ of Ar, i.e., between 11.0 and 11.4 eV, were used. Nevertheless, the absolute DCS values for Ar agree within mutual uncertainties as discussed in [42]. Since our normalization is based upon relative flow method, we prefer to use values from the most recent paper [42] and only for the energy of 250 eV, which is not available in [42] we used those from [43].

Since our experimental DCSs are obtained in the limited angular range, in order to obtained elastic integral (ICSs) and momentum transfer (MTCSs) cross-sections, our DCSs must be extrapolated to the smallest (0°) and the highest (180°) scattering angles. Our extrapolation takes into account the theoretically obtained shapes of DCSs (present model 2, at small (0–25°) and high (120–180°) scattering angles and present experimental values of DCSs near 25° and 120°. Thereafter, ICSs and MTCSs were obtained using extrapolated DCSs and appropriate integration defined as:

(14) $$ICS=2\pi \int_{0}^{\pi }DCS\left(\theta \right)\sin\theta d\theta$$

(15) $$MTCS=2\pi \int_{0}^{\pi }DCS\left(\theta \right)\left(1-\cos\theta \right)\sin\theta d\theta$$

The uncertainties of the relative DCSs consisted of statistical uncertainties and short-term stability uncertainties, caused by instability of the system. This uncertainty is increased by 20% for small scattering angles, due to the potential alteration of the interaction volume. Dominant uncertainties for absolute DCSs are those from reference cross-sections for Ar [42,43], and are taken to be about 20%. The DCS uncertainties obtained in such a manner, Δ, are presented in parenthesis within Table 2. The total uncertainties for absolute DCSs are about 30% for small angles and about 20% for the rest of the angular range. The total uncertainties of ICSs and MTCs arise from the DCSs uncertainties mentioned above and uncertainties of the extrapolation of DCSs to 0° and to 180° and numerical integration (10%).

## 4. Results

**Figure 1:**
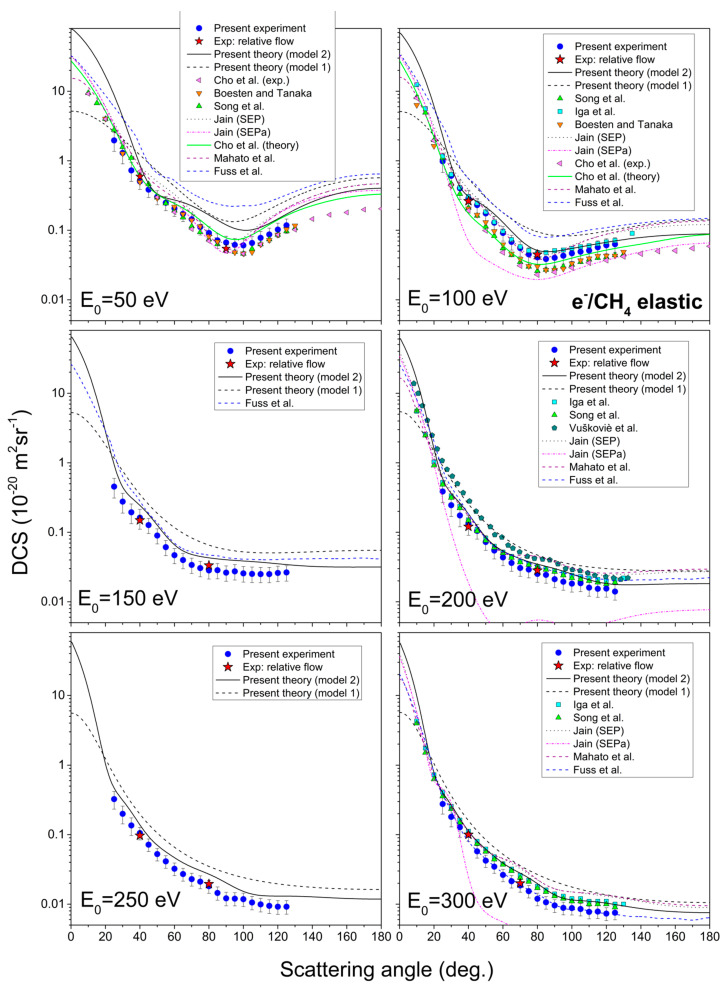
Angle differential cross-sections for elastic electron scattering from methane molecule, for six incident electron energies, from 50 eV to 300 eV. The present results include experiment (blue circles), relative flow absolute data (red stars), theory with molecular effects (full line) and simple sum theory (dashed line). Previous results, tabulated in Table 1, are also shown for the sake of comparison.

Measured results of the absolute differential cross-sections, integral cross-sections, and momentum transfer cross-sections, together with their corresponding uncertainties, are shown in Table 2. The results cover six incident electron energies, from 50 eV to 300 eV and angular range from 25° to 125°. In Figure 1, the present theoretical and experimental DCSs for all six incident electron energies are shown graphically. For the sake of comparison, other authors’ data [16,23,24,27,28,29,30,31], listed in Table 1, are shown in the same figure.

Theoretical results are shown in two approximations: the simple sum of individual atomic cross-sections (model 1, dashed black line) and with molecular effects taken into account (model 2, solid black line). It can be seen that those effects significantly modify DCSs. One of them, the absorption potential, plays a dominant role in this modification of cross-sections. In comparison with present experiment and other authors’ results, theory with included molecular effects showed better agreement, both qualitatively and quantitatively, as expected.

**Figure 2:**
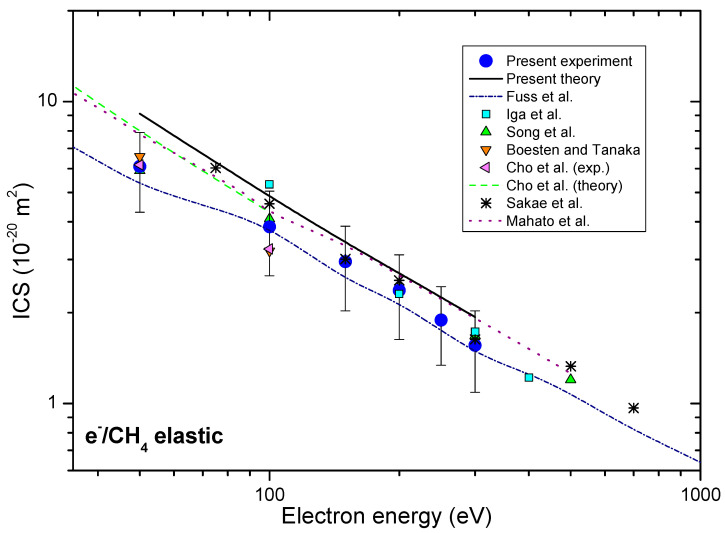
Integral cross-sections for elastic electron-methane collision, presented in energy range from 35 eV to 1000 eV. Present experimental (blue circles) and theoretical (solid black line) results are shown together with previous experimental, as well as the theoretical results, for comparison.

Obtained ICSs, together with other authors’ results [17,25,29,30,31,33,34], are presented in Figure 2.

## 5. Discussion

Experimental DCS at 50 eV exhibits wide minimum at 100° scattering angle, for which position and depth are in good agreement with other authors’ results [17,25,29,32,33], especially experimental ones [17,25,29]. Our calculations overestimate the measured DCS, but it matches with shape and is in a good agreement with other theories [29,32] at high scattering angles, above 110°. It is interesting to note that all existing experimental values at 50 eV impact energy agree among themselves within the estimated uncertainties. It is rather a problem with theories, which, in most cases, overestimate absolutes values or do so at certain angular ranges.

There are many experimental and theoretical data for DCS at 100 eV. This cross-section shows a wide and shallow local minimum at 80° scattering angle, which vanishes for incident electron energies above 150 eV. Its position is the same or similar for every shown result. Our measurement and calculation are in strong agreement with the experimental result of Iga et al. [31] and with the theories of Jain (SEP) [32] and Mahato et al. [33], except at higher scattering angles (from 105°). Other results are in good agreement with the shape, but quantitatively are underestimated in comparison with the present result. It seems that there are two classes of experimental values that differ in absolute values, one by Boesten and Tanaka [25] and Cho et al. [29] and the other by Iga et al. [31] and the present measurements. One of the significant differences between these two sets of data lie in the choice of the reference gas, the former used He while the later used Ne and Ar as a reference. Nevertheless the cross-sections for He are known with better accuracy than those of Ne and Ar, it seems plausible to conclude that more similar flow conditions between the reference gas and the target one give more reliable data. If it has been possible to obtain perfectly the same all conditions necessary for applying the relative flow method, then the choice of reference gas would be the one with the best-known cross-sections, i.e., He gas. However, since the method itself introduces additional uncertainties, in our opinion, it would be the best procedure to compare cross-sections with relatively similar flows within gas inlet system. 

As for DCSs for 150 eV, there are only results by Fuss et al. [34] available for comparison. At this energy, there are no local minima, like for 50 eV and 100 eV. Instead, there is a wide plateau from 70° scattering angle. Experiment and theory are in good agreement by the shape, but the experiment is, on average, about 30% quantitatively lower.

DCSs for 200 eV (both measured and calculated) show good agreement (within experimental uncertainty) with experimental results of Iga et al. [31], recommended data by Song et al. [17] and theoretical results by Jain (SEP) [32] and Mahato et al. [33] for smaller scattering angles (below 70°). Results by Vušković and Trajm ar [26] are good by shape but higher in absolute value.

For DCS for 250 eV incident electron energy, to the best of our knowledge, there are no published experimental or theoretical results. In the graph, it can be seen that our theory is slightly higher than the measured results, but both are similar qualitatively.

At 300 eV, the shapes and values of present theory and previous results by Iga et al. [31] and Song et al. [17] are in almost perfect agreement. The experimental result is just slightly lower on the absolute scale. Same as for every other energy, calculations by Jain [32] and Mahato et al. [33] agree good for smaller scattering angles, but for higher, they little overestimate other results.

The values of DCSs span over three and four orders of magnitude in presented energy range, what is the characteristic behavior for molecular targets and noticed also in the previous targets [42,48]. The general agreement among different experimental datasets and calculations is very good, and that is what one may expect at the present level of advanced experiments and sophisticated calculations [49].

The present experimental integral cross-sections ICSs are shown in Table 1 and together with other available results in Figure 2. Our experimental ICSs are placed between recommended Fuss et al. [34] and theoretical results Mahato et al. [33]. In the electron energy range from 50 eV to 300 eV most of the other experimental ICSs, like those by Boesten and Tanaka [25], Sakae et al. [30], Iga et al. [31], Song et al. [17], lie within the same uncertainty limits. Our experimental DCS data are obtained in rather limited angular range, and that is why the presented integral cross-sections depend strongly upon the extrapolation procedure. We have normalized our calculated DCS (model 2) to our measured absolute data to best match in shape and then used these values for integration. These values are presented in Table 2 with the uncertainty of 30% that arises from different plausible extrapolations. The absolute uncertainties of ICS and MTCS values are obtained from the difference of the corridor that is represented by maximal, DCS + Δ/2, and minimal DCS − Δ/2 values. 

**Figure 3:**
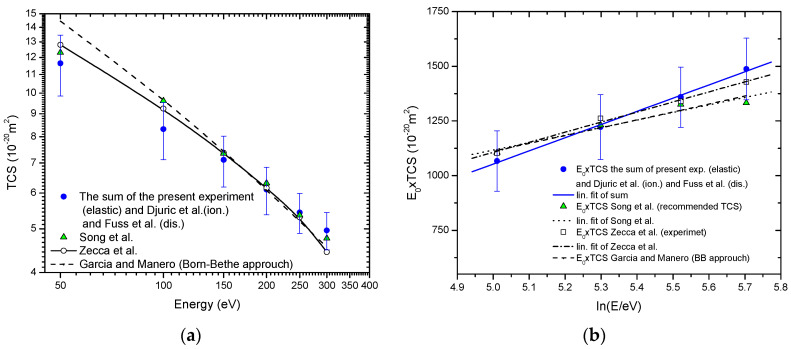
Total cross-sections for electron-methane collision in energy range from 45 eV to 300 eV. 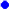, present; 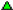, Song et al. (recommended values) [17]; 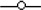, Zecca et al. (experiment) [51]; - - -, García and Manero (Born-Bethe approach) [52]: (**a**) Absolute values; (**b**) The Fano-Bethe plot: 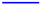, linear fit of present; • • •, linear fit of Song et al. [17]; – ∙ –∙ linear fit of Zecca et al. [51].

If total cross-section (TCS) we calculate as the sum of our measured ICSs, and ionization cross-sections obtained by Djurić et al. [50] plus the recommended neutral dissociation cross-sections by Fuss et al. [34], the TCS values obtained in such way agree well, within experimental uncertainties, with previously published experimental values by Zecca et al. [51] and recommended values by Song et al. [17]. Furthermore, for electron energies greater than 150 eV present TCS well agree with the semiempirical TCS by García and Manero [52] extrapolated in the interval energies of this paper. Total cross-sections for electron-methane collision in the energy range from 45 eV to 300 eV are contrasted in Figure 3a, while the corresponding Fano-Bethe plot is shown in Figure 3b. For electron energies from 150 eV to 300 eV Bethe plot of present TCS, References [17,51] and extrapolated semiempirical TCS García and Manero [52], agree within the limits of experimental uncertainties. This, under the given conditions, suggests that Born-Bethe approximation is valid in the electron energy range from 150 eV to 300 eV.

## 6. Conclusions

In order to provide the insight into methane/electron interaction, measurements and calculations of elastic electron scattering from methane target were performed. To summarize, calculated and measured cross-sections for elastic CH_4_-electron scattering in (50–300) eV incident electron energy range are reported and, conceivably, they will serve as a dependable standard for related investigations in the future. Good agreement between present theory and experiment and results available in the literature is noticed. Also, it is shown that molecular effects (especially absorption effects) play a crucial role on calculated cross-sections, particularly for very small scattering angles (below 20°). The experiment was performed in two independent steps. First was measuring relative DCSs for fixed electron energy in function of scattering angle. Second was obtaining pair of absolute points for every energy, by relative flow method and Ar as reference gas. These absolute points were then used for normalization of relative DCSs. These independent results agree well, which is confirmation of the reliability of the experimental method. DCSs for the incident energy 250 eV is given without previous results known to the authors.

Nevertheless there are many studies of electron elastic scattering by methane molecule, our impression is that the present study is important to pinhole the absolute cross-sections, to confirm recommended sets of data for this process given by Song et al. [17] and to bring new results at one impact energy. Last but not the least, we stress the problem of the choice of a reference gas in the relative flow measurements and the necessity of choosing gases with similar flowing conditions. That could bring methane molecule to be a new standard for cross-sections measurements of other hydrocarbons or larger organic molecules.

## Figures and Tables

**Table 1:** List of experimental and theoretical work on differential cross-section (DCS) for elastic scattering of electrons from methane molecule, covering energy range from 50 to 300 eV.

Authors	Experiment Type with Normalization Method/Theoretical Approach	Energy Range (eV)	Angular Range (°)
Boesten and Tanaka [25]	Crossed beams, simultaneous measurements of DCS of He	1.5–100	10–130
Vušković and Trajmar [26]	Crossed beams, normalized to other authors results	20–200	8–130
Cho et al. [29]	Crossed beams, relative flow (He)/Schwinger variational method	5–100	10–180
Sakae et al. [30]	Crossed beams, relative flow (He)	75–700	5–135
Iga et al. [31]	Crossed beams, relative flow (Ne)/Schwinger variational method	100–500/	10–135
		1–500	
Jain [32]	Spherical optical complex potential model	0.1–500	0–180
Mahato et al. [33]	Gaussian wave functions	10–500	0–180
Fuss et al. [34]	Optical potential method and the independent atom approximation including the screen corrected additivity rule (SCAR)	0.7–10,000	0–180

**Table 2:** Experimental DCSs, integral cross-sections (ICSs), and momentum transfer cross-sections (MTCSs) for elastic scattering of electrons from methane molecule with absolute uncertainties Δ given in parentheses with 2 last digits. Values from 25° to 125° are measured and uncertainties arise from both statistical and short-term stability and include absolute uncertainties of cross-sections for reference Ar gas. Values from 0° to 20° and from 125° to 180° are extrapolated values and used to calculate ICSs and MTCSs. Uncertainties at small scattering angles are estimated to be 30%.

θ (°)	DCS (10^−20^ m^2^sr^−1^)					
	50 (eV)	100 (eV)	150 (eV)	200 (eV)	250 (eV)	300 (eV)
0	17.5(5.3)	19.3(5.9)	25.1(7.9)	24.0(7.4)	24.5(7.0)	23.4(6.6)
5	13.8(4.1)	13.2(4.0)	15.8(4.7)	13.5(4.1)	12.9(3.9)	11.0(3.3)
10	9.8(2.9)	7.9(2.5)	7.9(2.5)	6.1(1.9)	5.0(1.4)	3.9(1.1)
15	6.3(1.9)	3.9(1.2)	3.2(1.0)	2.43(0.76)	1.50(43)	1.07(30)
20	3.7(1.1)	1.70(53)	1.09(35)	0.83(0.26)	0.43(12)	0.435(78)
25	1.96(60)	0.98(30)	0.45(14)	0.39(12)	0.325(93)	0.277(78)
30	1.30(40)	0.61(19)	0.277(87)	0.246(76)	0.200(57)	0.181(51)
35	0.73(22)	0.40(12)	0.194(61)	0.175(54)	0.136(39)	0.128(36)
40	0.50(12)	0.288(89)	0.162(51)	0.132(41)	0.106(22)	0.100(20)
45	0.384(88)	0.230(55)	0.127(31)	0.105(25)	0.072(15)	0.058(12)
50	0.302(70)	0.174(41)	0.090(22)	0.072(17)	0.053(11)	0.0423(85)
55	0.249(57)	0.128(30)	0.061(15)	0.055(13)	0.0413(85)	0.0347(70)
60	0.206(47)	0.091(22)	0.047(12)	0.044(10)	0.0324(67)	0.0264(53)
65	0.173(40)	0.068(16)	0.0398(99)	0.0362(88)	0.0271(57)	0.0214(43)
70	0.144(33)	0.055(13)	0.0340(84)	0.0306(74)	0.0229(48)	0.0186(38)
75	0.111(26)	0.0451(11)	0.0305(76)	0.0291(71)	0.0211(44)	0.0154(31)
80	0.092(21)	0.0409(98)	0.0283(70)	0.0252(61)	0.0181(38)	0.0120(25)
85	0.072(17)	0.0381(93)	0.0284(71)	0.0244(59)	0.0145(31)	0.0107(22)
90	0.067(16)	0.0403(96)	0.0263(66)	0.0212(52)	0.0121(26)	0.0096(20)
95	0.062(14)	0.043(10)	0.0274(68)	0.0195(48)	0.0120(26)	0.0088(18)
100	0.061(14)	0.047(11)	0.0256(64)	0.0183(45)	0.0118(26)	0.0088(18)
105	0.066(15)	0.049(12)	0.0252(63)	0.0185(45)	0.0106(23)	0.0085(18)
110	0.078(18)	0.051(12)	0.0250(62)	0.0160(39)	0.0099(22)	0.0078(16)
115	0.087(20)	0.057(14)	0.0250(62)	0.0154(38)	0.0094(21)	0.0078(16)
120	0.102(24)	0.061(15)	0.0260(65)	0.0155(38)	0.0092(20)	0.0073(15)
125	0.118(27)	0.063(15)	0.0264(66)	0.0141(35)	0.0092(20)	0.0075(16)
130	0.136(31)	0.066(16)	0.0258(63)	0.0139(35)	0.0092(20)	0.0072(15)
140	0.176(41)	0.071(17)	0.0249(61)	0.0140(35)	0.0091(20)	0.0066(14)
150	0.230(53)	0.074(18)	0.0244(61)	0.0142(35)	0.0089(20)	0.0061(13)
160	0.311(72)	0.077(18)	0.0243(61)	0.0144(36)	0.0086(19)	0.0059(12)
170	0.44(10)	0.079(19)	0.0243(61)	0.0144(36)	0.0084(19)	0.0058(12)
180	0.67(15)	0.079(19)	0.0243(61)	0.0145(36)	0.0083(19)	0.0057(12)
ICS’s	6.1(1.8)	3.8(1.2)	2.95(0.92)	2.37(0.74)	1.89(0.55)	1.56(0.47)
MTCS’s	2.09(0.53)	0.98(0.26)	0.45(0.13)	0.33(0.10)	0.222(0.054)	0.177(0.043)

## Data Availability

The data presented in this study is contained within the aricle.

## Abbreviations

R. F.	radio frequency
PECVD	plasma-enhanced chemical vapor deposition
DCSs	differential cross-sections
ICSs	integral cross-sections
MTCSs	momentum transfer cross-sections
SCAR	screen corrected additivity rule
ELSEPA	Dirac partial-wave calculation of elastic scattering of electrons and positrons by atoms, positive ions and molecules
DCMA	double cylindrical mirror analyzer
RFM	relative flow method
TCS	total cross-section
